# Functionalities of electronic routine health information systems related to newborn data: findings of the IMPULSE study in Uganda, Ethiopia, Tanzania, and the Central African Republic

**DOI:** 10.7189/jogh.15.04330

**Published:** 2025-12-05

**Authors:** Mary Ayele, Ilaria Mariani, Firehiwot Abathun, Ousman Mouhamadou, Jacqueline Minja, Muhumuza Kananura Rornald, Francesca Tognon, Louise Tina Day, Johan Sæbø, Henok Fisseha, Felix Bundala, Paolo Dalena, Sara Geremia, Lorenzo Giovanni Cora, Joy E Lawn, Giovanni Putoto, Peter Waiswa, Donat Shamba, Marzia Lazzerini

**Affiliations:** 1 Doctors with Africa CUAMM, Addis Ababa, Ethiopia; 2 Institute for Maternal and Child Health IRCCS Burlo Garofolo, WHO Collaborating Centre for Maternal and Child Health, Trieste, Italy; 3 Doctors with Africa CUAMM, Bangui, Central African Republic; 4 Department of Health Systems, Impact Evaluation and Policy, Ifakara Health Institute, Tanzania; 5 Demographic Dynamic and Population Health Unit, African Population and Health Research Center, Dakar, Senegal; 6 Doctors with Africa CUAMM, Padua, Italy; 7 London School of Hygiene & Tropical Medicine London, UK; 8 University of Oslo, Oslo, Norway; 9 Addis Ababa City Administration Health Bureau, Addis Ababa, Ethiopia; 10 Institute for Maternal and Child Health IRCCS Burlo Garofolo, Trieste, Italy; 11 University of Trieste, Trieste, Italy; 12 Makerere University of Public Health, Kampala, Uganda

## Abstract

**Background:**

Adequate functionality of electronic routine health information systems (eRHISs) is crucial for data use, yet few studies explored it in relation to newborn and stillbirth data in Africa.

**Methods:**

We conducted this cross-sectional study between November 2022 and July 2024 in data offices at central and subnational levels in 12 regions and 4 city administrations in the Central African Republic (CAR), Ethiopia, Tanzania, and Uganda. Except for end-user perspectives (collected *via* interviews), we collected data related to eRHIS functionalities by direct observation following standard operating procedures as for the Every Newborn-Measurement Improvement for Newborn & Stillbirth Indicators (EN-MINI) Tool 3.1, based on the Performance of Routine Information System Management (PRISM) framework. We analysed data according to the PRISM Users’ Kit.

**Results:**

We assessed 53 data offices in total. All countries used the same software application, the District Health Information Software 2 (DHIS2). Settings were heterogeneous across countries, with a tendency for DHIS2 to offer fewer functionalities to users in the CAR. Overall functionalities for generating facility annual summary reports (100% in all countries) and for calculating percentage of reports received/expected (75.0% in Ethiopia to 88.9% in Tanzania) were widely available. Data integration and data disaggregation, meanwhile, had lower availability. Functionalities for calculating coverage on specific indicators, such as respectful care, were lacking in all countries, those for quality assurance varied across countries, while those related to data visualisation were almost always available in Uganda and Tanzania, but showed specific gaps in Ethiopia (*i.e.* for early initiation breastfeeding), and most often lacked in the CAR. Most end-users indicated needs for eRHIS improvement (ranging from 37.5% in Ethiopia to 100% in the CAR; *P* = 0.001), with 17.0% reporting needs for major improvement (from 10.0% in Uganda to 28.6% in the CAR; *P* = 0.001). Subgroup analyses suggested high within-country heterogeneity and more eRHIS functionalities available at central *vs*. subnational level.

**Conclusion:**

Identified strengths and gaps in existing DHIS2 functionalities can inform the design of context-specific interventions that will enhance data use for reducing neonatal mortality and stillbirth rates.

Each year, around 2.3 million infants die within their first 28 days of life, with 1.9 million being stillborn [1,2]. Nearly all of these fatalities – approximately 98% – happen in low- and middle-income countries (LMICs) [1–4]. An additional estimated one million newborns who are small and ill manage to survive, but experience long-term disabilities and severe health issues such as cerebral palsy, cognitive impairment, chronic lung disease, and impaired vision and hearing [5,6]. Their families also face long-term psychological and financial challenges, which, in turn, can negatively impact the newborns’ health [5,6]. Consequently, a significant amount of human potential for lifelong health and well-being is lost due to both newborn mortality and morbidity [5–7].

It is estimated that around 30 million newborns worldwide need some form of inpatient care each year. Babies who have special needs, such as those being born prematurely or at a small size for their gestational age, or those with birth defects or postnatal infections, often require hospitalisation for a significant duration [7]. Ensuring that all newborns receive optimal care is crucial for their survival and is considered a fundamental human right [7,8]. In recent years, there has been growing recognition of the importance of delivering high-quality care to newborns within health care facilities. Despite this, numerous studies conducted in both low- [9–12] and high-income countries [13–16] have identified notable gaps in the quality of newborn care, indicating persistent disparities in the provision thereof [13–16].

Accelerating change requires improvements in the availability and use of routine data in order to enhance service coverage, quality and outcomes, as well as to promote accountability and action [3,5,7]. In this sense, significant changes are required to achieve the targets outlined in the World Health Organisation (WHO) Survive, Thrive, and Transform agenda [7]. Addressing gaps in the availability and use of newborn and stillbirth data is essential to ending preventable deaths (*i.e.* to surviving), ensuring good health and well-being (*i.e.* to thriving), and revolutionising the care provided to small and sick new-borns (*i.e.* to transforming) [7]. Prioritising, standardising, and improving data flow is also one of the key strategic actions to accelerate progress towards Sustainable Development Goals, as envisioned in several initiatives, such as the Every Newborn Action Plan & Ending Preventable Maternal Mortality, recently renamed as the Every Woman Every Newborn Everywhere, chaired by WHO, United Nations Children’s Fund, United Nations Population Fund, and the Child Survival Action Initiative [17].

The functionality of electronic routine health information systems (eRHISs) – defined as how well they are designed for the task they are expected to perform [18] – is critical for enabling informed decision-making, ensuring high-quality care to newborns and their parents, and reducing neonatal mortality and stillbirth rates [5,7,19,20]. These eRHISs have the potential to support quality healthcare in LMICs by providing quality data [21], supporting disease surveillance and outbreak response [22], facilitating clinical workflows [23] and strengthening overall health systems by supporting planning, resource allocation, and decision-making [24].

The District Health Information Software 2 (DHIS2) is the most widely used eRHIS software. Aside from shared functionalities, it is highly customisable, offers several configurations (*i.e.* (not) activating certain functionalities), and allows for personalisation of user access rights according to different user roles. However, few studies have documented the functionalities of eRHISs in African countries [24], including key aspects such as the availability of functionalities to calculate summary reports and coverage for newborn indicators, quality assurance functionalities, data integration, data disaggregation, interoperability across different systems, and end-user perspective [25].

The IMProving qUaLity and uSE of newborn measures (IMPULSE) is a collaborative project among different institutions – the London School of Hygiene & Tropical Medicine in UK, the WHO Collaborating Center for Maternal and Child Health in Italy, the Doctors with Africa *Collegio Universitario Aspiranti Medici Missionari* (*CUAMM*), the Ifakara Health Institute in Tanzania, and Makerere University in Uganda – that aims to address some of these gaps, with a current focus on four African countries. This paper is part of a journal collection, including other papers reporting on related aspects, such as on newborn and stillbirth data quality [26], data use [27], eRHIS usability [28], organisational aspects [29] and availability of resources. The whole supplement utilises the PRISM framework (Appendix S1 in the **Online Supplementary Document**), a conceptual model of the health information system performance elaborating the various determinants at input, processes, output, and outcome levels. Here we report on the IMPULSE study findings related to the observed functionalities of eRHISs, collected using a standardised tool and methodology across four countries and at different health system levels, including district/regional health offices and four central offices/ministries of health (MoHs).

## METHODS

### Study design and participants

Both the IMPULSE and this study were cross-sectional in design, so we reported our findings per the STROBE checklist [30] (Appendix S2 in the **Online Supplementary Document**).

We collected data across 12 regions and 4 city administrations of the Central African Republic (CAR), Ethiopia, Tanzania, and Uganda (Appendix S3 in the **Online Supplementary Document**). We selected regions based on a balance of the following three criteria:

1. heterogeneity, *i.e.* regions with different characteristics, including underperforming for maternal and neonatal mortality, hard-to-reach areas, or humanitarian settings;

2. regions where the implementing agency, Doctors with Africa *CUAMM* had an office/project that could facilitate coordination or other or other easy-to-reach regions (well-connected or near to the capital city);

3. regions prioritised per request of the local MoH.

The IMPULSE project had predefined criteria for sampling, as detailed elsewhere [26,27]. Briefly, it focussed on facilities providing at least newborn essential care, *i.e.* exclusively on comprehensive emergency obstetric and newborn care health facilities, with or without neonatal inpatient care, and all related data offices. A fixed number of facilities in each category was pre-defined for each region, and within this rule, facilities with the higher number of deliveries were selected. We also selected all data office receiving data from the selected facilities, at either district, regional (if relevant, as regional data office exist only in Ethiopia and Tanzania), and central levels/MoHs. In accordance with the related PRISM tools, we focussed selectively on variables collected only at data office level, as functionalities of the eRHIS in most African countries are set up at national or regional/district level, and do not differ at facility level. Each data office usually supervises several facilities.

### Data collection tools

We collected data between 22 November 2022 and 10 July 2024 using the EN-MINI PRISM tools, a tested adaptation of the PRISM framework for newborn and stillbirth data that includes ready-to-use digital data collection tools [18].

This set of tools has been developed by the Every Newborn – Birth Indicators Research Tracking in Hospitals phase 2 research partners and further optimised (version 2) and field tested during the start-up phase of the IMPULSE study, in collaboration with the IMPULSE international advisory board and the four IMPULSE national advisory boards (one for each country) [31]. The tools were originally developed in English and Swahili, and were translated by IMPULSE native speaking experts (either medical doctors or data experts) into Amharic (for use in Ethiopia) and French (for use in the CAR) before the start of IMPULSE data collection. The tools were also back translated to better assess the accuracy of the translation.

Here we describe data collected with the EN-MINI-PRISM Tool 3.1, dedicated to assessing the functionality of the eRHIS at data office level [18]. Eighty-eight variables were collected mostly by direct observation. Users’ perspectives on needs for improvements were also collected on selected variables, from the reference person in each office (usually the individual responsible for the health management information systems at central levels/MoHs, district health officers at district levels; and regional/provincial health officers at regional/provincial health offices), according to the EN-MINI-PRISM standard operating procedures [18]. Measures for data quality, completeness, timeliness, and usability are presented elsewhere [26,28].

### Data quality assurance

Teams of 3–6 data collectors with previous experience in health information system data collection who were fluent in local languages (Swahili, Amharic, French) collected data in each country under the supervision of experienced study coordinators (FA, JM, MA, MKR, OM). Besides standard EN-MINI-PRISM Tools training materials, training for both data collectors and study coordinators included field practices; a series of preliminary meetings to clarify any doubt questions and answers; a file where all questions and answers were recorded; a WhatsApp group to solve any remaining question in real-time.

Data collection was done following instructions embedded in the EN-MINI-PRISM tools. Additional details were clarified through standard operating procedures files for data collection, developed in dialogues with study coordinators. We collected data using password-protected tablets or phones using an Open Data Kit-based secure digital platform and uploaded them to a secure, encrypted server hosted by SurveyCTO (Dobility, Inc., Washington, D.C., USA) through a community license [32]. The platform for data entry included checks for data completeness and plausibility. Region and site name and key informant role were collected, but no individual identifiers. Data were downloaded in each country, checked to ensure they did not include any key informant identifiers, and securely transferred to the study group.

We also developed, tested, and regularly used a pre-defined monitoring and evaluation Excel file reviewed by a supervisor (FT) to keep track of data timeliness, completeness, and progression of data collection. We discussed any missing or implausible data in real-time.

During the first months of data collection, independent data analysts (IM, PD, SG) conducted four rounds of interim analyses to check for data completeness, internal consistency, plausibility. Results were discussed after each round, and data collection was adjusted accordingly (*e.g.* if there were missing data).

We cross-checked the results of the final analysis against those of the automated EN-MINI-PRISM analysis tool [31], and we held data validation workshops in each country to discuss IMPULSE study phase 1 findings and their implications.

### Data analysis

We analysed data in accordance with the pre-defined analytical plan outlined in the PRISM User’s Kit 2019 [32]. For additional variables not included in the PRISM manual, data were analysed according a pre-defined plan of analysis.

We summarised all variables descriptively, reporting the results as absolute numbers and percentages for each relevant variable. We reported frequencies for the overall sample and by each country and performed χ^2^ test or Fisher’s exact test, as appropriate, to test whether they differed across countries. We likewise conducted exploratory subgroup analyses to explore frequencies at different health system levels (central levels/MoHs compared to subnational, *i.e.* regional or district data offices) and by each region in each country.

A two tailed *P*-value <0.05 was considered statistically significant. We performed all statistics in *R*, version 4.1.1 (R Foundation for Statistical Computing, Vienna, Austria).

### Ethical aspects

We performed all data collection according to the General Data Protection Regulation. We did not gather any information that could disclose participants’ identity, thereby ensuring their anonymity. Data was transmitted and stored in password protected tablets, after which it was uploaded into encrypted servers. Paper documents were stored in locked filing cabinets. Most data were collected by direct observation, except for staff interviews. Staff participating in interviews were informed of the study objectives and methods, as well as their rights in declining participation, by way of an information sheet prior to enrolling, and each individual provided written consent before responding to the survey.

## RESULTS

### Characteristics of the sample

We included 53 health data offices across the four African countries. Forty-two (79.2%) were at the district level, seven (13.2%) at the regional level (only in Ethiopia and Tanzania), and four (7.5%) at the central/MoH data offices (one in each country) (**Table 1**; Appendix S4 in the **Online Supplementary Document**). All countries used the DHIS2 as their eRHIS.

**Table 1:** IMPULSE study: PRISM administrative unit, presented as n (%)

	Overall (n = 53)	CAR (n = 7)	Ethiopia (n = 8)	Tanzania (n = 18)	Uganda (n = 20)	
District health office	42 (79.2)	6 (85.7)	3 (37.5)	14 (77.8)	19 (95.0)	
Regional/provincial health office	7 (13.2)	0 (0.0)	4 (50.0)	3 (16.7)	0 (0.0)	
Central ministry of health	4 (7.5)	1 (14.3)	1 (12.5)	1 (5.6)	1 (5.0)	

CAR – Central African Republic

### Existing eRHIS functionalities

**Figure 1:**
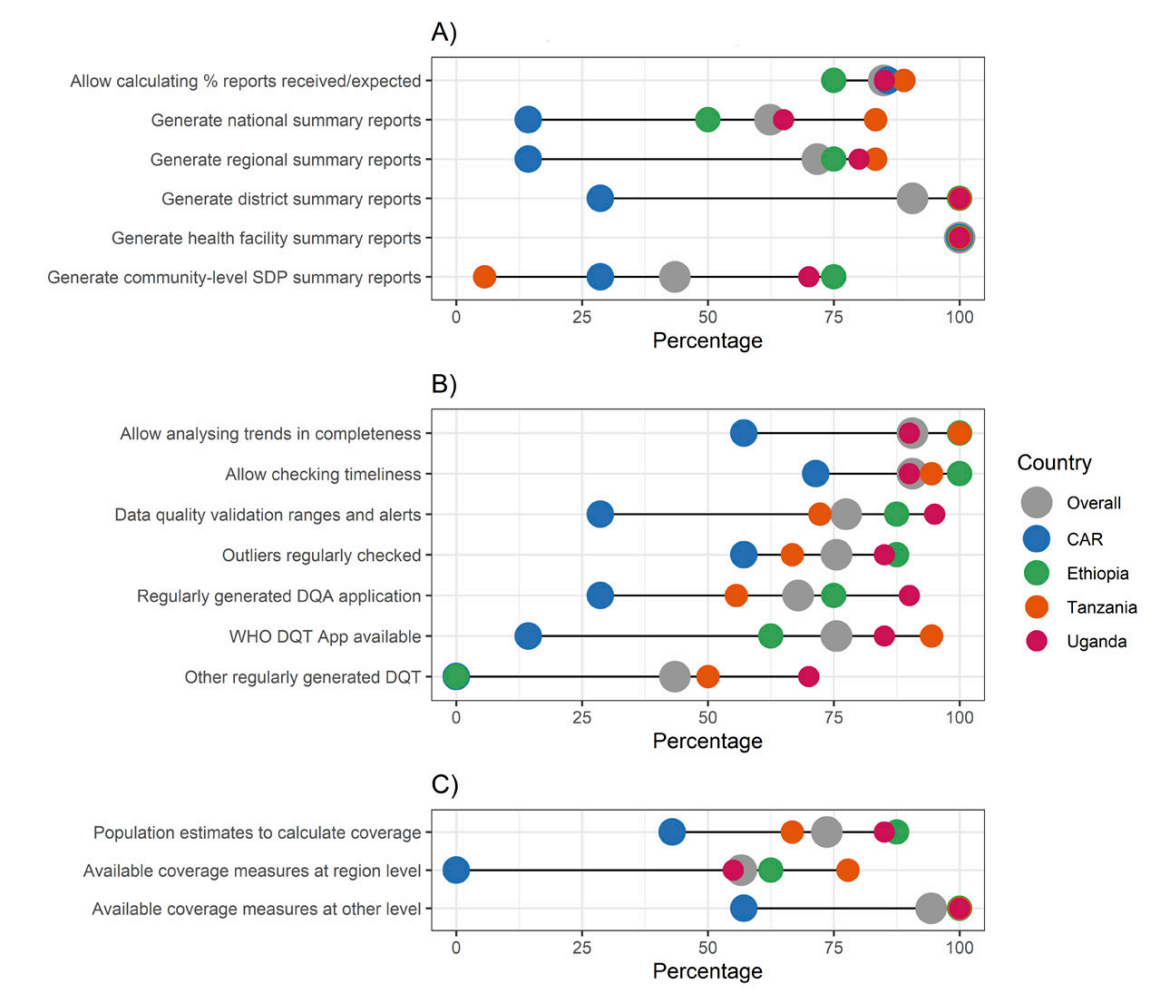
IMPULSE study – existing eRHIS functionalities. Each dot represents the value for one country; differences in dot size are only due to the need to graphically represent overlapping percentages. **Panel A.** Generating summary reports. **Panel B.** Ensuring data quality. **Panel C.** Calculating coverage. **Panel D.** Capacity development. CAR – Central African Republic, DQA – data quality assessment, DQT – data quality tool, eRHIS – electronic routine health information system, IDSR – integrated disease surveillance and response, SDP – service delivery point.

**Figure 2:**
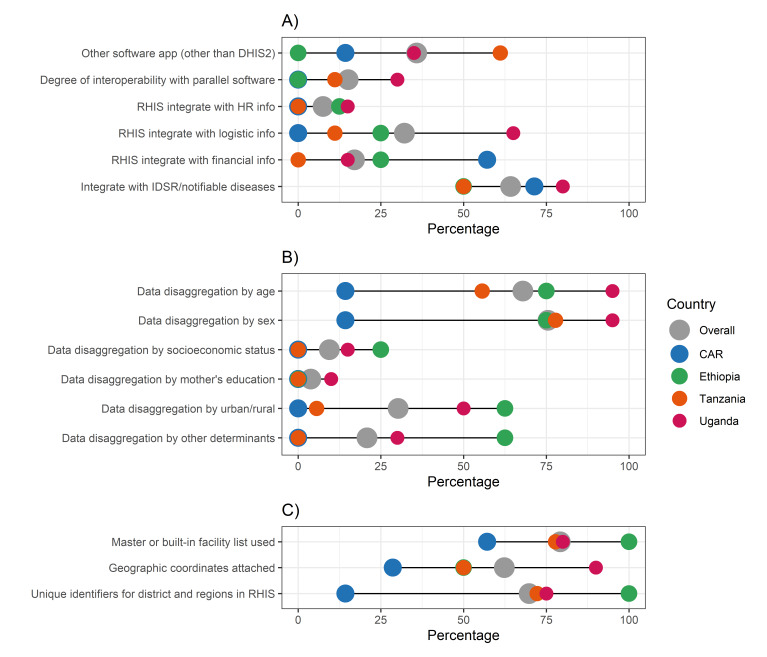
IMPULSE study – existing eRHIS functionalities. Each dot represents the value for one country; differences in dot size are only due to the need to graphically represent overlapping percentages. **Panel A.** Data integration. **Panel B.** Data disaggregation. **Panel C.** Unique identifier. CAR – Central African Republic, DHIS2 – District Health Information System 2, eRHIS – electronic routine health information system, HR – human resources.

**Figure 3:**
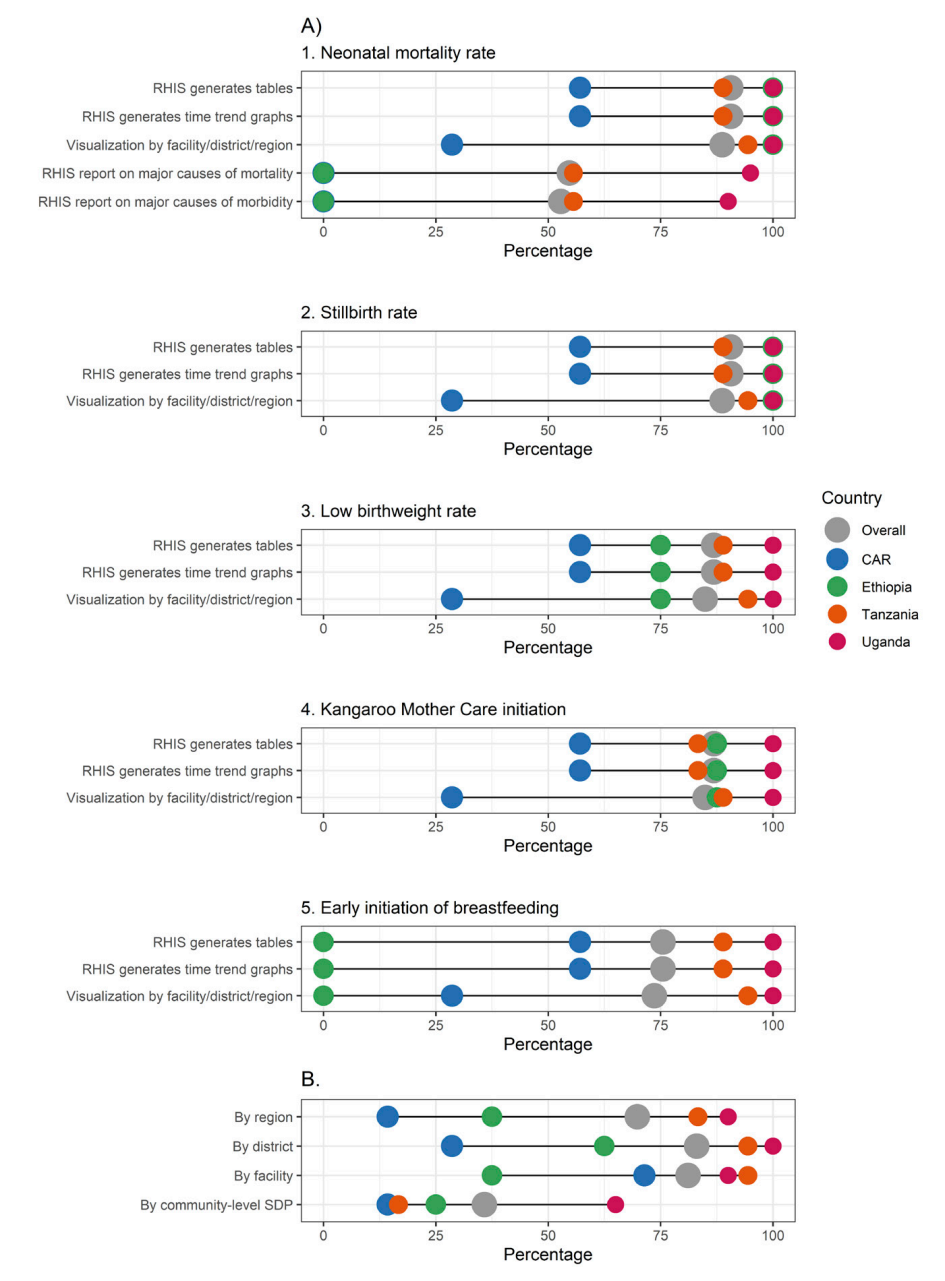
IMPULSE study – existing eRHIS functionalities: data visualisation. Each dot represents the value for one country; differences in dot size are only due to the need to graphically represent overlapping percentages. **Panel A.** Visualisation by type of indicator. **Panel B.** Visualisation by thematic map. CAR – Central African Republic, eRHIS – electronic routine health information system, SDP – service delivery point.

We observed eRHIS settings to be very heterogenous for all functionalities explored across countries, with a tendency for the eRHIS in Ethiopia, Uganda, and Tanzania to have higher functionalities than the one in the CAR (**Figure 1**, **Figure 2**, **Figure 3**; Appendix S5–11 in the **Online Supplementary Document****)**.

Existing functionalities to generate summary reports were heterogenous across regions (**Figure 1**, Panel A; Appendix S5 in the **Online Supplementary Document**). From one side, all assessed data offices hosted functionalities for generating health facility annual summary reports (n = 53, 100%), with the existing settings allowing for the calculation of the percentage of reports received/expected (75.0% in Ethiopia to 88.9% in Tanzania) and for generating district annual reports (28.6% in the CAR to 100% in Ethiopia, Tanzania, and Uganda) at very high percentages in most countries. Conversely, functionalities to generate regional (14.3% in the CAR to 83.3% in Tanzania), national (14.3% in the CAR to 83.3% in Tanzania), and community level (5.6% in Tanzania to 75.0% in Ethiopia) annual reports were less frequent and more heterogeneous across countries.

Availability of quality assurance functionalities varied across countries, with data from Uganda and Ethiopia, followed by Tanzania, generally showing the higher frequency of the desired functionalities, and those for the CAR showing the lower frequencies (**Figure 1**, Panel B; Appendix S6 in the **Online Supplementary Document**). Differences across countries were particularly wide for some items such as data quality validation ranges and alerts (28.6% in the CAR to 95.0% in Uganda; *P* = 0.004); presence of a regularly generated data quality assessment application (28.6% in the CAR to 90.0% in Uganda; *P* = 0.009); availability of the DHIS2 WHO data quality tool (14.3% in the CAR to 94.4% in Tanzania; *P* < 0.001) or other regularly generated data quality tools (from 0% in the CAR to 70.0% in Uganda; *P* < 0.001).

We noted variations in the functionalities available to calculate coverage indicators across the countries. Population estimates to calculate coverage were available overall in 39 data offices (73.6%), ranging from 42.9% in the CAR to 87.5% in Ethiopia (*P* = 0.129) (**Figure 1**, Panel C; Appendix S7 in the **Online Supplementary Document**). Functionalities to calculate coverage for selected indicators varied (overall percentages above 80% for 5 out of 17 key indicators) and showed specific gaps: in the CAR, for example, functionalities for calculating the coverage indicator related to hospital neonatal mortality rate was lacking (0%), as did the coverage for the indicator for breastfeeding within one hour from birth in Ethiopia (0%). Moreover, functionalities for calculating coverage for the following indicators were generally low: antenatal corticosteroid use (0.0% in the CAR to 50.0% in Uganda; *P* = 0.041), companion of choice during birth (0.0% in the CAR, Ethiopia, and Tanzania to 20.0% in Uganda; *P* = 0.130), zero separation of mother and newborn (0.0% in the CAR, Ethiopia, and Tanzania, to 20.0% in Uganda; *P* = 0.130), and respectful care indicators (0% in CAR, Ethiopia, and Tanzania to 15.0% in Uganda; *P* = 0.319).

Approximately one third (n = 19, 35.8%) of the sites overall, particularly in Tanzania (n = 11, 61.1%) and Uganda (n = 7, 35.0%), implemented other software in parallel to DHIS2, such as programme-specific software (*e.g.* vaccinations, mortality audits, performance-based financing) or software related to research projects. However, there was low interoperability between the existing parallel systems and the DHIS2 (n = 8, 15.1%) (**Figure 2**, Panel A; Appendix S8 in the **Online Supplementary Document**). Data integration with the disease surveillance and response system (overall: n = 34, 64.2%) ranged from 50.0% in Tanzania and Ethiopia to 80.0% in Uganda (*P* = 0.209). Other data integration functionalities were lacking overall, with low country frequencies for eRHIS integration with human resources information system (0.0% in the CAR and Tanzania to 15.0% in Uganda; *P* = 0.303), logistics information systems (0% in the CAR to 65.0% in Uganda; *P* = 0.001), and financial information (0.0% in Tanzania to 57.1% in the CAR; *P* = 0.005).

Overall, functionalities for capturing disaggregating data by age (14.3% in the CAR to 95.0% in Uganda; *P* < 0.001) and sex (14.3% in the CAR to 95.0% in Uganda; *P* < 0.001) varied by country (**Figure 2**, Panel B; Appendix S9 in the **Online Supplementary Document**). Other functionalities for data disaggregation were poorly available, such as those for socioeconomic status (0.0% in the CAR and Tanzania to 25.0% in Ethiopia; *P* = 0.130), maternal education (0.0% in the CAR, Ethiopia, and Tanzania to 10.0% in Uganda; *P* = 0.739), urban/rural setting (0.0% in the CAR to 62.5% in Ethiopia; *P* = 0.001) and other determinants of health (0.0% in the CAR and Tanzania to 62.5% in Ethiopia; *P* = 0.001).

Regarding functionalities that enabled the acquisition of a unique identifier (identify facilities/units), we noted fair availability for the study variables overall, although again with major variability across countries. Most (57.1% in the CAR to 100% in Ethiopia; *P* = 0.237) of the assessed health offices had a master or built-in facility list used, which was kept updated (**Figure 2**, Panel C; Appendix S10 in the **Online Supplementary Document**).

Functionalities on newborn data visualisation by type of indicator **(****Figure 3**, Panel A; Appendix S11 in the **Online Supplementary Document**) were almost always available in Uganda and Tanzania, showed specific gaps in Ethiopia for the indicator of early initiation breastfeeding, and most often lacked in the CAR. An eRHIS functionality to generate reports on major causes of both neonatal mortality and morbidity was lacking in the CAR and Ethiopia (0.0%), available in half of Tanzanian data offices (55.6% for both neonatal mortality and morbidity), and was highly available in Uganda (95.0% and 90.0% for neonatal mortality and morbidity, respectively; *P* < 0.001).

Functionalities of visualisations by thematic maps (**Figure 3**, Panel B; Appendix S11 in the **Online Supplementary Document**) were again highly variable across countries. There was a better practice of visualisation data using thematic maps at district (82.4%) and facility (80.4%) *vs*. region (68.6%) and community (33.3%) levels. The CAR was an exception, as it had better functionalities at facility (71.4%) *vs*. district (28.6%), region (14.3%), and community (14.3%) levels.

### Subgroup analyses

Subgroup analyses by region showed large heterogeneity within each country (Appendix S5–11 in the **Online Supplementary Document**), particularly for quality assurance functionalities. For example, data quality validation ranges and alerts were set in 0.0% to 50.0% of data offices in the CAR, 50.0% to 100% of data offices in Ethiopia, 20.0% to 100% of data offices in Uganda, and 40.0% to 100% data offices in Tanzania. Low frequencies in data visualisation were encountered in the CAR regions, in Oromia, Ethiopia, for low birthweight rate, and in Iringa, Tanzania for kangaroo mother care initiation.

Desired functionalities were more often available at central/MoH levels than at subnational data offices (Appendix S5–11 in the **Online Supplementary Document**). Data at the central/MoHs levels showed some gaps in data integration, data disaggregation, and functionalities for calculate coverage in all countries, with less functionalities noted in the CAR. In Ethiopia, data visualisation *via *thematic maps was lacking at central/MoH levels, while in Tanzania, no summary report community level was available at central/MoH levels.

### End-users’ perspectives

Overall, 100% of staff in the CAR, 94.4% in Tanzania, 65.0% in Uganda, 37.5% in Ethiopia (*P* = 0.001) indicated a need for improvement in the eRHIS (Appendix S12 in the **Online Supplementary Document**). Approximately 28.6% of staff in the CAR, 25.0% in Ethiopia, 16.7% Tanzania, and 10.0% in Uganda (*P* = 0.001) reported a need for a major improvement in the eRHIS.

## DISCUSSION

This study generated new evidence on functionalities of the eRHIS from data collected in 12 regions and 4 city administrations in four African countries (**Box 1**). Overall, we found eRHIS functionalities to be heterogenous both across and within countries. There was a trend for the DHIS2 in Uganda, followed by the one in Ethiopia and Tanzania, to provide more functionalities than the DHIS2 in the CAR, and for data offices at central levels/MoHs to have more functionalities than at the sub-national level. No country was totally free from gaps, although all showed particular strengths in some assessed variables. End-user perspectives aligned with results from assessments on observed data, suggesting that some improvements were needed.

Box 1 **Key study findings**

What was known before this study?

– Newborn and stillbirth healthcare data are needed for action to end preventable mortality and morbidity and reach globally agreed goals by 2030.

– Availability and use of newborn and stillbirth data are directly connected to the existence of adequate eRHIS functionalities.

– Little is known regarding existing functionalities of the eRHIS related to newborn and stillbirth data from health data offices in the CAR, Ethiopia, Tanzania, and Uganda.

What did we find and what does it mean?

– Data offices in the CAR, Ethiopia, Tanzania, and Uganda used the same eRHIS software application, the DHIS2.

– eRHIS functionalities varied across and within country, and were significantly lower in the CAR than elsewhere.

– Common strengths in all countries were functionalities for generating facility annual summary reports and for calculating percentage of reports received/expected, while common gaps were found in data integration and data disaggregation.

– eRHIS improvements requested by end-users were in line with the objectively assessed functionalities.

– Other IMPULSE publications are reporting on staff performance on using eRHIS, on human resources, and on newborn data quality and use.

What novelties does this study bring?

– First four-country multi-regional study in Africa to explore the eRHIS functionalities related to newborn and stillbirth data in high mortality settings, utilising a standardised methodology and many variables.

What is next for implementation?

– Optimising eRHIS functionalities, particularly data integration, to streamline newborn and stillbirth data quality and use is essential for monitoring and reducing neonatal mortality and stillbirth rates.

What research gaps remain?

– Further research on reasons for differences among countries and levels of the health system can establish priorities for future actions.

– The PRISM methodology could be further optimised, particularly by identifying aggregate indicators that can be used in multivariate analyses to identify factors related to best/worst performance.

Our results can be explained by several factors. First, the EN-MINI PRISM tools define functionality as a wide construct which covers characteristics inherent to the software itself, the configurations of the software in the four included countries, and the authorities of various users to use these functionalities to complete desired tasks. Differences in timings and geographical coverage of implementation, as well in DHIS2 customisation – which allows for demand-driven applications and modules to be adapted to specific country needs, local workflows, and user preferences [33,34] – may all contribute to explaining the heterogeneity we observed here, as pointed out by previous reviews [35,36]. The DHIS2 is a common digital application used for eRHISs in Africa; it was adopted in Uganda in January 2011 [37], in Tanzania in December 2013 [38,39], in Ethiopia in 2019 [33] and sub-nationally in the CAR in 2019 [40], with major benefits on data quality and use [33,35,37]. However, all districts in the CAR were connected to DHIS2 only in mid-2023 [41]. Moreover, although data collectors directly observed functionalities and were trained and experienced in the related data collection methods, we cannot entirely exclude that some of the gaps observed in existing functionalities could be related to a knowledge gap among staff at data office levels. Another IMPULSE paper reports detailed data on staff competences in using the eRHIS [28] and similarly showed large heterogeneity across countries and levels in the health system. Gaps in training of staff, as well in key resources such as internet access, were also documented by the IMPULSE project and will be reported elsewhere, as will other related technical, organisational, and management factors [29].

There is limited evidence related to eRHIS functionalities in African countries, in particular when related to newborn health. A literature review of research from 11 LMICs countries summarised the overall strengths and operational challenges of the DHIS2, highlighting that its implementation improved the creation of charts and reports, visual data analysis through user-defined dashboards, geographic information system interfaces, and data integration with other e-health systems, as well as quality, timeliness, and completeness of the data, although the availability of high-quality, complete data was reported to be problematic in some countries or regions [35]. Qualitative studies in Ethiopia and Tanzania reported suboptimal use of mapping functionalities and data validation tools of DHIS2 [38,42]. Lack of data disaggregation within the DHIS2 has been reported by other studies, such as in the context of vaccine surveillance in Uganda [43].

Concerning digital innovation, some countries are moving forward new functionalities rather quickly. For example, Ethiopia, besides having recently incorporated the Ethiopian calendar (which has 13 months and is different from Gregorian calendar) in DHIS2, also integrated on-the-spot data quality checks and enhanced graphical user interface [35]. However, other aspects of data integration, such as harmonisation of indicators (*e.g.* early breastfeeding initiation) with international standards, still require further attention in Ethiopia, as in other countries [44]. In their scoping review, Byrne and Sæbø recommended a need for more detailed documentation of how key indicators such as coverage are calculated [45]. A mixed-method study conducted in three Ethiopian health facilities identified 33 major eRHIS functionalities – like event report, event visualise, league table, and maps – and reported that most were underused, misused, or unknown to system users [46]. The presence of eRHIS functionalities, therefore, may not translate into higher data quality without the end-users’ knowledge and uptake thereof [46,47]. Another paper of the IMPULSE project will report on user abilities in utilising the eRHIS [28]. Further high-quality research should be conducted on eRHIS functionalities related to newborn and stillbirth data in other countries.

In regard to end-users’ perspective, perceived needs for eRHIS improvement mostly align with the objectively assessed functionalities. Ethiopia’s efforts for country adaptation may explain the lower needs for eRHIS improvement reported by its end-users compared to those in other countries. Other studies suggest that end-users appreciated DHIS2’s user-friendly interface, offline functionalities, and potentiality for data digitalisation [42,48–50]. We also collected end-user suggestion on how to improve the eRHIS functionalities within the IMPULSE project, and will report on these qualitative data elsewhere.

Our findings call for action to address the identified gaps on the eRHIS functionalities related to newborn data. This could be critical for providing high-quality newborn and stillbirth data, and potentially essential for monitoring and reducing neonatal mortality and stillbirth rates [18]. We suggest investigating potential for interoperability across different eRHIS systems as a key area to solve some of the identified challenges and improve functionalities for data disaggregation. Interoperability and data integration are central priority for harmonising key priorities and for improving the unification of data collection and management systems at both national and subnational levels, the importance of which has been recognised in WHO and national recommendations [51–54]. Improving interoperability/data integration across different reporting platform will provide policy makers with a comprehensive overview of newborn and stillbirth data, especially after integrating health data with financial and logistic data. Improved interoperability could also reduce staff workload [55], particularly in settings where multiple redundant paper-based forms and electronic tools coexist.

The DHIS2 has been conceived to allow flexibility and data integration: its architecture enables connectivity with other external software, configurations adaptable to national health system needs, the collection, monitoring and sharing of standardised data (including disaggregated data), as well as other functionalities [35,56,57]. The modular nature of the DHIS2 creates spaces for local participatory design, which could lead to better fit to local use practices [58]. Monitoring the availability of eRHIS functionalities when compared to the needs of health professionals and health system, as well their actual use in the routine work, are critical aspect highlighted elsewhere [33,35]. Other aspects beyond eRHIS functionalities likewise need to be addressed to ensure adequate data quality and use, as for the PRISM framework [31,32], including a good infrastructure, fast internet connection and stable electricity, adequate number of staff, regular training, constructive and effective supervision and other feedback systems, sustainable programmes, effective governance mechanisms at all levels, and political support [19,20,33,35,38,42,46,49]. In particular, the heterogeneity in the DHIS2 functionalities we observed here suggests a need to strengthen accountability and governance systems at all levels to minimise gaps and disparities.

We acknowledge that our findings cannot be generalised to the whole countries. The limited sample size, the sampling criteria, and security issues in the CAR and Ethiopia may have introduced selection bias. We also cannot completely rule out observer bias. However, we note that our study is the first assessment specifically related to functionalities of eRHIS relevant to newborn data in Africa. It included data on 88 variables, reflecting the complexity of the system. The assessment was conducted using a standardised methodology previously applied in many countries [17,32] and adapted for the assessment of stillbirth and newborn data quality [8,59]. We collected data through direct observation while implementing several data quality assurance procedures. The study also included diverse geographical areas, as well as different levels and types of data offices. 

The EN-MINI-PRISM tool 3.1 does not collect sociodemographic information for end-users [18]. Furthermore, while we collected data during the COVID-19 pandemic, we note that other studies reported on eRHIS functionalities expanding during this period [60,61]. Another limitation is the lack of a summary dependent variable recognised by PRISM framework, which discouraged us from utilising regression models. Future studies could further improve EN-MINI-PRISM tools by identifying a summary variable for each tool that can be used in multivariate analyses to identify factors related to best/worst performance, ensuring collection of end-users’ sociodemographic data, and providing a more precise operational definition of functionality – ideally separated from structural capacity, human factors, or organisational determinants. Despite these limitations, our findings can drive actions tailored for improving eRHIS functionalities, and the EN-MINI-PRISM assessment can be easily replicated over time (as well as in other sites) to further check progress. 

## CONCLUSIONS

We explored existing eRHIS functionalities related to newborn and stillbirth data across several geographical regions in Uganda, Ethiopia, Tanzania, and the CAR. We highlighted both strengths and gaps in all the four countries, with more gaps noted in the CAR than elsewhere, providing the basis for further action and context-specific interventions. Further studies should identify reasons for the differences among countries and health system levels, as their finding could help with targeting future actions. Ensuring good eRHIS functionalities can enhance the quality, processing, and use of newborn and stillbirth data, with the final aim of improving the quality of newborn care services and reducing neonatal mortality and stillbirth rates.

## Additional material


Online Supplementary Document
