# Supplementary material for: Functionalities of electronic routine health information systems related to newborn data: findings of the IMPULSE study in Uganda, Ethiopia, Tanzania, and the Central African Republic
Source: J Glob Health. 2025 Dec 5;15:04330. doi: 10.7189/jogh.15.04330 (PMC12677248; doi:10.7189/jogh.15.04330)
Supplement: Online Supplementary Document [file jogh-15-04330-s001.pdf]

Supplement to: Ayele M, Mariani I, Abathun A, Mouhamadou O, Minja J, Kananura RM, Tognon F, Day LT, Sæbø J, Fisseha H, Bundala F, Dalena P, Geremia S, Cora LG, Lawn JE, Putoto G, Waiswa P, Shamba D, Lazzerini M. Functionalities of electronic routine health information systems related to newborn data: findings of the IMPULSE study in Uganda, Ethiopia, Tanzania, and the Central African Republic. J Glob Health. 2025;15:04330.

Table of contents

Appendix S1. PRISM conceptual framework 2

Appendix S2. The Strengthening the Reporting of Observational Studies (STROBE) Checklist 3

Appendix S3. Geographical distribution of the regions included in the IMPULSE study 5

Appendix S4. Characteristics of the sample 6

Appendix S5. Existing eRHIS functions: generating summary reports 7

Appendix S6. Existing eRHIS functions: ensuring data quality 9

Appendix S7. Existing eRHIS functions: calculating coverage 10

Appendix S8. Existing eRHIS functions: data integration (all OBSERVED) 12

Appendix S9. Existing eRHIS functions: data disaggregation 13

Appendix S10. Existing eRHIS functions: unique identifier 14

Appendix S11. Existing eRHIS functions: data visualization 15

Appendix S12. End-Users' perspectives 17

Appendix S1. PRISM conceptual framework

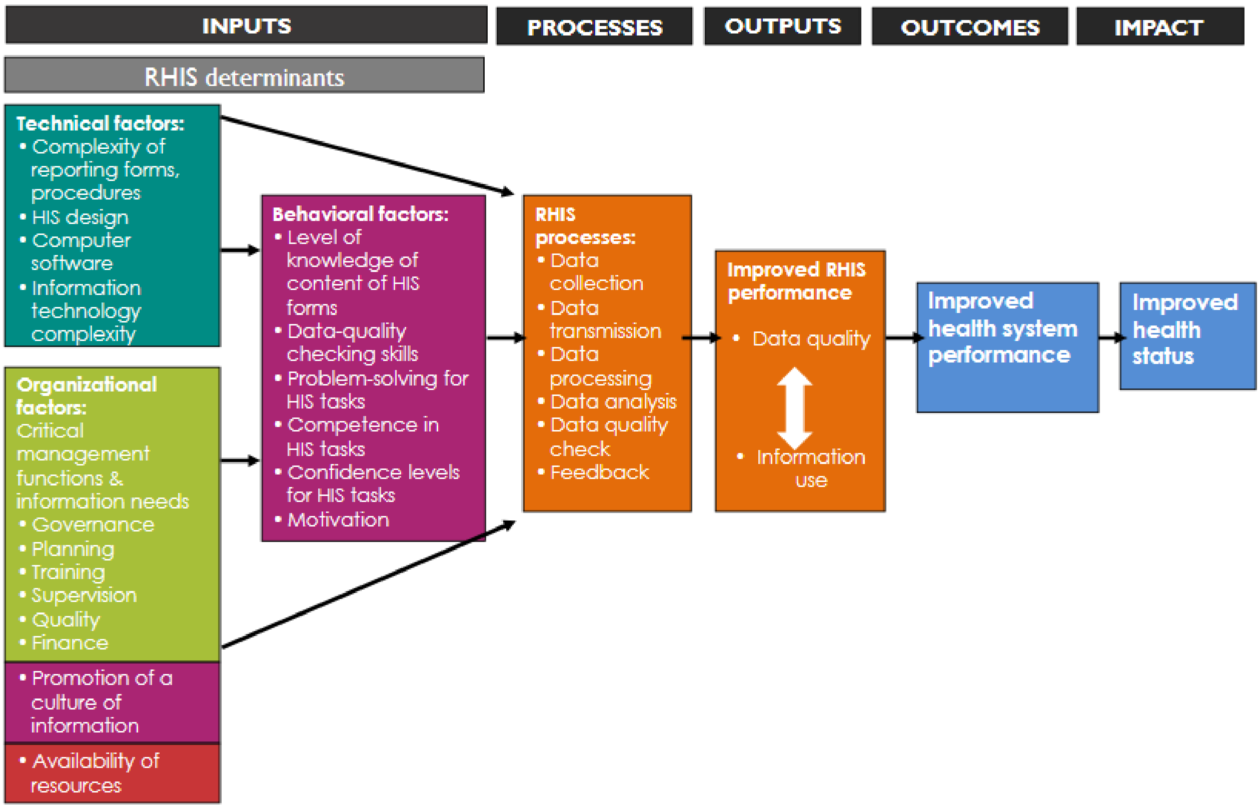

Reference: Aqil A, Lippeveld T, Hozumi D. PRISM framework: a paradigm shift for designing, strengthening and evaluating routine health information systems. Health Policy Plan. 2009;24:217–28. doi:10.1093/heapol/czp010

Abbreviations: PRISM = Performance of Routine Information System Management

Appendix S2. The Strengthening the Reporting of Observational Studies (STROBE) Checklist

	Item No	Recommendation	Pages
**Title and abstract**	1	(*a*) Indicate the study's design with a commonly used term in the title or the abstract	4
		(*b*) Provide in the abstract an informative and balanced summary of what was done and what was found	4
**Introduction**			
Background/rationale	2	Explain the scientific background and rationale for the investigation being reported	6-7
Objectives	3	State specific objectives, including any prespecified hypotheses	7
**Methods**			
Study design	4	Present key elements of study design early in the paper	7
Setting	5	Describe the setting, locations, and relevant dates, including periods of recruitment, exposure, follow-up, and data collection	7-8
Participants	6	(*a*) Give the eligibility criteria, and the sources and methods of selection of participants	7
Variables	7	Clearly define all outcomes, exposures, predictors, potential confounders, and effect modifiers. Give diagnostic criteria, if applicable	8-9
Data sources/ measurement	8*	For each variable of interest, give sources of data and details of methods of assessment (measurement). Describe comparability of assessment methods if there is more than one group	7-8
Bias	9	Describe any efforts to address potential sources of bias	8-9
Study size	10	Explain how the study size was arrived at	7-8
Quantitative variables	11	Explain how quantitative variables were handled in the analyses. If applicable, describe which groupings were chosen and why	8
Statistical methods	12	(*a*) Describe all statistical methods, including those used to control for confounding	9
		(*b*) Describe any methods used to examine subgroups and interactions	9
		(*c*) Explain how missing data were addressed	-
		(*d*) If applicable, describe analytical methods taking account of sampling strategy	-
		(*e*) Describe any sensitivity analyses	-
**Results**			

Participants	13*	(a) Report numbers of individuals at each stage of study—eg numbers potentially eligible, examined for eligibility, confirmed eligible, included in the study, completing follow-up, and analysed	9-10
		(b) Give reasons for non-participation at each stage	-
		(c) Consider use of a flow diagram	-
Descriptive data	14*	(a) Give characteristics of study participants (eg demographic, clinical, social) and information on exposures and potential confounders	10
		(b) Indicate number of participants with missing data for each variable of interest	-
Outcome data	15*	Report numbers of outcome events or summary measures	10-12
Main results	16	(*a*) Give unadjusted estimates and, if applicable, confounder-adjusted estimates and their precision (eg, 95% confidence interval). Make clear which confounders were adjusted for and why they were included	10-12
		(*b*) Report category boundaries when continuous variables were categorized	-
		(*c*) If relevant, consider translating estimates of relative risk into absolute risk for a meaningful time period	-
Other analyses	17	Report other analyses done—eg analyses of subgroups and interactions, and sensitivity analyses	12
**Discussion**			
Key results	18	Summarise key results with reference to study objectives	12
Limitations	19	Discuss limitations of the study, taking into account sources of potential bias or imprecision. Discuss both direction and magnitude of any potential bias	14
Interpretation	20	Give a cautious overall interpretation of results considering objectives, limitations, multiplicity of analyses, results from similar studies, and other relevant evidence	12-14
Generalisability	21	Discuss the generalisability (external validity) of the study results	12-14
**Other information**			
Funding	22	Give the source of funding and the role of the funders for the present study and, if applicable, for the original study on which the present article is based	15

Notes: * Give information separately for exposed and unexposed groups.

Appendix S3. Geographical distribution of the regions included in the IMPULSE study

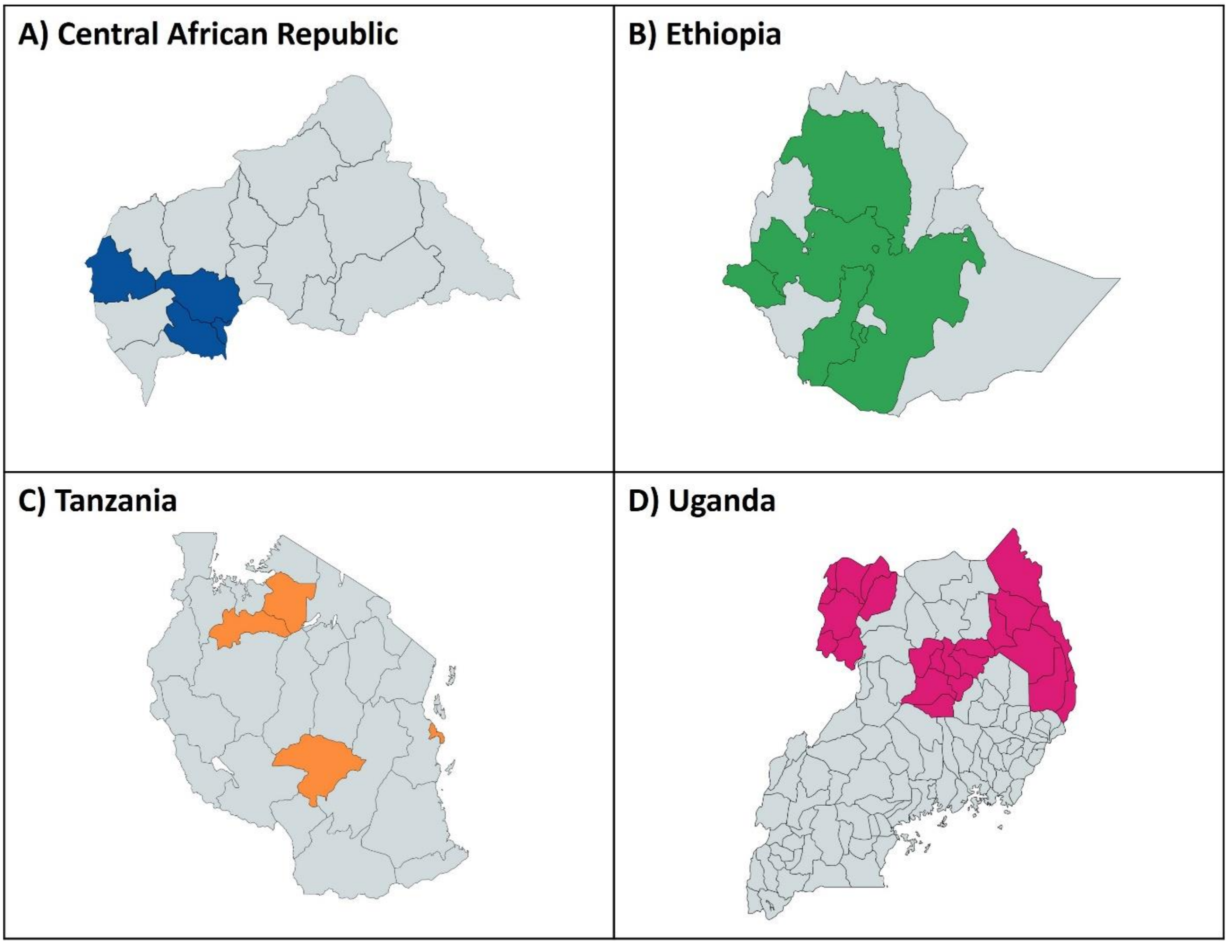

Notes: the figure shows regions included in the IMPULSE study. For Central African Republic: Bangui City Administration and Health region 1, 2 and 7; for Ethiopia: Addis Ababa City Administration, Oromia, Amhara and Gambella, South Ethiopia and Sidama; for Tanzania: Dar es Salaam City Administration, Iringa, Shinyanga, Simiyu; for Uganda: Lango, Karamoja, West-Nile, Kampala City Administration.

Appendix S4. Characteristics of the sample

Question	Answer	Overall		CAR		Ethiopia		Tanzania		Uganda		p-value
		N=53		N=7		N=8		N=18		N=20		
		n	%	n	%	n	%	n	%	n	%	
PRISM admninistrative unit	District Health Office	42	79.2	6	85.7	3	37.5	14	77.8	19	95.0	0.007
	Regional / provincial health office	7	13.2	0	0	4	50	3	16.7	0	0	
	Central MOH	4	7.5	1	14.3	1	12.5	1	5.6	1	5.0	
By Country and Region	**CAR**											<0.001
	Bangui City Administration	1	1.9	1	14.3	0	0	0	0	0	0	
	Health region 1	1	1.9	1	14.3	0	0	0	0	0	0	
	Health region 2	2	3.8	2	28.6	0	0	0	0	0	0	
	Health region 7	3	5.7	3	42.9	0	0	0	0	0	0	
	**Ethiopia**											
	Addis Ababa City Administration	2	3.8	0	0	2	25	0	0	0	0	
	Amhara and Gambella	2	3.8	0	0	2	25	0	0	0	0	
	Oromia	3	5.7	0	0	3	37.5	0	0	0	0	
	South Ethiopia and Sidama	1	1.9	0	0	1	12.5	0	0	0	0	
	**Tanzania**											
	Dar es Salaam City Administration	1	1.9	0	0	0	0	1	5.6	0	0	
	Iringa	6	11.3	0	0	0	0	6	33.3	0	0	
	Shinyanga	5	9.4	0	0	0	0	5	27.8	0	0	
	Simiyu	6	11.3	0	0	0	0	6	33.3	0	0	
	**Uganda**											
	Kampala City Administration	1	1.9	0	0	0	0	0	0	1	5	
	Lango	6	11.3	0	0	0	0	0	0	6	30	
	Karamoja	8	15.1	0	0	0	0	0	0	8	40	
	West-Nile	5	9.4	0	0	0	0	0	0	5	25	

Abbreviations: CAR = Central African Republic; MOH=ministry of health

Appendix S5. Existing eRHIS functions: generating summary reports

Number of the question	Question	Answer	Overall		CAR								TOTAL		Ethiopia										TOTAL		Tanzania								TOTAL		Uganda								TOTAL		P-value by country
			N=53		Bangui City Administration (central/MOH) N=1		Health region 1 N=1		Health region 2 N=2		Health region 7 N=3		N=7		Addis Ababa City Administration N=2		Addis Ababa City (central/MOH) N=1		Amhara and Gambella N=2		Oromia N=3		South Ethiopia and Sidama N=1		N=8		Dar es Salaam City Administration (central/MOH) N=1		Iringa N=6		Shinyanga N=5		Simiyu N=6		N=18		Kampala City Administration (central/MOH) N=1		Lango N=6		Karamoja N=8		West-Nile N=5		N=20		
			n	%	n	%	n	%	n	%	n	%	n	%	n	%	n	%	n	%	n	%	n	%	n	%	n	%	n	%	n	%	n	%	n	%	n	%	n	%	n	%	n	%	n	%	
ESF_010.	Allowing calculating % reports received/expected (observed)	Yes	45	84.9	1	100	0	0	2	100	3	100	6	85.7	2	100	1	100	2	100	2	66.7	0	0	6	75	1	100	4	66.7	5	100	6	100	16	88.9	1	100	6	100	6	75	4	80	17	85	0.894
ESF013_1a.	Generates summary reports (1. National A. Monthly)	Yes	33	62.3	1	100	0	0	0	0	0	0	1	14.3	1	50	1	100	1	50	1	33.3	1	100	4	50	1	100	3	50	5	100	6	100	15	83.3	1	100	5	83.3	3	37.5	4	80	13	65	0.011
ESF013_1b.	Generates summary reports (1. National B. Quarterly)	Yes	33	62.3	1	100	0	0	0	0	0	0	1	14.3	1	50	1	100	1	50	1	33.3	1	100	4	50	1	100	3	50	5	100	6	100	15	83.3	1	100	5	83.3	3	37.5	4	80	13	65	0.011
ESF013_1c.	Generates summary reports (1. National C. Annually)	Yes	33	62.3	1	100	0	0	0	0	0	0	1	14.3	1	50	1	100	1	50	1	33.3	1	100	4	50	1	100	3	50	5	100	6	100	15	83.3	1	100	5	83.3	3	37.5	4	80	13	65	0.011
ESF013_1d.	Generates summary reports (1. National D. Customized reporting period)	Yes	33	62.3	1	100	0	0	0	0	0	0	1	14.3	1	50	1	100	1	50	1	33.3	1	100	4	50	1	100	3	50	5	100	6	100	15	83.3	1	100	5	83.3	3	37.5	4	80	13	65	0.011
ESF013_2a.	Generates summary reports (2. Regional A. Monthly)	Yes	38	71.7	1	100	0	0	0	0	0	0	1	14.3	2	100	1	100	2	100	1	33.3	1	100	6	75	1	100	3	50	5	100	6	100	15	83.3	1	100	5	83.3	5	62.5	5	100	16	80	0.006
ESF013_2b.	Generates summary reports (2. Regional B. Quarterly)	Yes	38	71.7	1	100	0	0	0	0	0	0	1	14.3	2	100	1	100	2	100	1	33.3	1	100	6	75	1	100	3	50	5	100	6	100	15	83.3	1	100	5	83.3	5	62.5	5	100	16	80	0.006
ESF013_2c.	Generates summary reports (2. Regional C. Annually)	Yes	38	71.7	1	100	0	0	0	0	0	0	1	14.3	2	100	1	100	2	100	1	33.3	1	100	6	75	1	100	3	50	5	100	6	100	15	83.3	1	100	5	83.3	5	62.5	5	100	16	80	0.006
ESF013_2d.	Generates summary reports (2. Regional D. Customized reporting period)	Yes	38	71.7	1	100	0	0	0	0	0	0	1	14.3	2	100	1	100	2	100	1	33.3	1	100	6	75	1	100	3	50	5	100	6	100	15	83.3	1	100	5	83.3	5	62.5	5	100	16	80	0.006
ESF013_3a.	Generates summary reports (3. District A. Monthly)	Yes	48	90.6	1	100	0	0	1	50	0	0	2	28.6	2	100	1	100	2	100	3	100	1	100	8	100	1	100	6	100	5	100	6	100	18	100	1	100	6	100	8	100	5	100	20	100	<0.001
ESF013_3b.	Generates summary reports (3. District B. Quarterly)	Yes	48	90.6	1	100	0	0	1	50	0	0	2	28.6	2	100	1	100	2	100	3	100	1	100	8	100	1	100	6	100	5	100	6	100	18	100	1	100	6	100	8	100	5	100	20	100	<0.001
ESF013_3c.	Generates summary reports (3. District C. Annually)	Yes	48	90.6	1	100	0	0	1	50	0	0	2	28.6	2	100	1	100	2	100	3	100	1	100	8	100	1	100	6	100	5	100	6	100	18	100	1	100	6	100	8	100	5	100	20	100	<0.001
ESF013_3d.	Generates summary reports (3. District D. Customized reporting period)	Yes	44	83	1	100	0	0	0	0	0	0	1	14.3	2	100	1	100	2	100	3	100	1	100	8	100	1	100	4	66.7	5	100	6	100	16	88.9	1	100	6	100	7	87.5	5	100	19	95	<0.001
ESF013_4a.	Generates summary reports (4. Health facility A. Monthly)	Yes	53	100	1	100	1	100	2	100	3	100	7	100	2	100	1	100	2	100	3	100	1	100	8	100	1	100	6	100	5	100	6	100	18	100	1	100	6	100	8	100	5	100	20	100	>0.99
ESF013_4b.	Generates summary reports (4. Health facility B. Quarterly)	Yes	53	100	1	100	1	100	2	100	3	100	7	100	2	100	1	100	2	100	3	100	1	100	8	100	1	100	6	100	5	100	6	100	18	100	1	100	6	100	8	100	5	100	20	100	>0.99
ESF013_4c.	Generates summary reports (4. Health facility C. Annually)	Yes	53	100	1	100	1	100	2	100	3	100	7	100	2	100	1	100	2	100	3	100	1	100	8	100	1	100	6	100	5	100	6	100	18	100	1	100	6	100	8	100	5	100	20	100	>0.99
ESF013_4d.	Generates summary reports (4. Health facility D. Customized reporting period)	Yes	48	90.6	1	100	0	0	1	50	3	100	5	71.4	2	100	1	100	2	100	3	100	1	100	8	100	1	100	4	66.7	5	100	6	100	16	88.9	1	100	6	100	7	87.5	5	100	19	95	0.271
ESF013_5a.	Generates summary reports (5. Community-level SDP A. Monthly)	Yes	21	39.6	1	100	0	0	0	0	1	33.3	2	28.6	1	50	1	100	2	100	2	66.7	1	100	6	75	0	0	1	16.7	0	0	0	0	1	5.6	1	100	6	100	2	25	3	60	12	60	<0.001
ESF013_5b.	Generates summary reports (5. Community-level SDP B. Quarterly)	Yes	25	47.2	1	100	0	0	0	0	1	33.3	2	28.6	1	50	1	100	2	100	2	66.7	1	100	6	75	0	0	1	16.7	0	0	0	0	1	5.6	1	100	6	100	5	62.5	4	80	16	80	<0.001

ESF013_5c.	Generates summary reports (5. Community-level SDP C. Annually)	Yes	23	43.4	1	100	0	0	0	0	1	33.3	2	28.6	1	50	1	100	2	100	2	66.7	1	100	6	75	0	0	1	16.7	0	0	0	0	1	5.6	1	100	6	100	3	37.5	4	80	14	70	<0.001
ESF013_5d.	Generates summary reports (5. Community-level SDP D. Customized reporting period)	Yes	22	41.5	1	100	0	0	0	0	1	33.3	2	28.6	1	50	1	100	2	100	2	66.7	1	100	6	75	0	0	1	16.7	0	0	0	0	1	5.6	1	100	6	100	3	37.5	3	60	13	65	<0.001

Abbreviations: CAR= Central African Republic; MOH=ministry of health; SDP = service delivery point

Appendix S6. Existing eRHIS functions: ensuring data quality

Number of the question	Question	Answer	Overall		CAR								Total		Ethiopia										Total		Tanzania								Total		Uganda								Total		P-value by country
			N=53		Bangui City Administration (central/MOH) N=1		Health region 1 N=1		Health region 2 N=2		Health region 7 N=3		N=7		Addis Ababa City Administration N=2		Addis Ababa City (central/MOH) N=1		Amhara and Gambella N=2		Oromia N=3		South Ethiopia and Sidama N=1		N=8		Dar es Salaam City Administration (central/MOH) N=1		Iringa N=6		Shinyanga N=5		Simiyu N=6		N=18		Kampala City Administration (central/MOH) N=1		Lango N=6		Karamoja N=8		West-Nile N=5		N=20		
			n	%	n	%	n	%	n	%	n	%	n	%	n	%	n	%	n	%	n	%	n	%	n	%	n	%	n	%	n	%	n	%	n	%	n	%	n	%	n	%	n	%	n	%	
ESF_011	Allow analysing trends in completeness (observed)	Yes	48	90.6	1	1	0	0	0	0	3	100	4	57.1	2	100	1	100	2	100	3	100	1	100	8	100	1	100	6	100	5	100	6	100	18	100	1	100	6	100	6	75	5	100	18	90.0	0.010
ESF_012	Allow checking timeliness (observed)	Yes	48	90.6	1	1	0	0	1	50	3	100	5	71.4	2	100	1	100	2	100	3	100	1	100	8	100	1	100	5	83.3	5	100	6	100	17	94.4	1	100	6	100	6	75	5	100	18	90.0	0.321
ESF_023.01	Data quality validation ranges and alerts	Yes	41	77.4	1	1	0	0	0	0	1	33.3	2	28.6	1	50	0	0	2	100	3	100	1	100	7	87.5	1	100	4	66.7	2	40	6	100	13	72.2	1	100	6	100	7	87.5	5	100	19	95.0	0.004
ESF_023.02	Outliers regularly checked	Yes	40	75.5	1	1	0	0	1	50	2	66.7	4	57.1	1	50	0	0	2	100	3	100	1	100	7	87.5	1	100	4	66.7	1	20	6	100	12	66.7	1	100	6	100	5	62.5	5	100	17	85.0	0.331
ESF_023.1	Regularly generated DQA application (observed) (yes=Monthly,Quarterly,Annually,Other)	Yes	36	67.9	1	1	0	0	0	0	1	33.3	2	28.6	2	100	1	100	1	50	2	66.7	1	100	6	75	1	100	4	66.7	2	40	3	50	10	55.6	1	100	6	100	6	75	5	100	18	90.0	0.009
ESF_023.3	WHO DQT App available (observed)	Yes	40	75.5	0	0	0	0	1	50	0	0	1	14.3	0	0	0	0	2	100	2	66.7	1	100	5	62.5	1	100	5	83.3	5	100	6	100	17	94.4	1	100	4	66.7	7	87.5	5	100	17	85.0	<0.001
ESF_023.4	Other regularly generated DQT (observed) (yes=Monthly,Quarterly,Annually,Other)	Yes	23	43.4	0	0	0	0	0	0	0	0	0	0	0	0	0	0	0	0	0	0	0	0	0	0	1	100	4	66.7	2	40	2	33.3	9	50	1	100	4	66.7	5	62.5	4	80	14	70.0	<0.001

Abbreviations: CAR= Central African Republic; DQA = Data Quality Assessment; DQT = Data Quality Tool; MOH=ministry of health

Appendix S7. Existing eRHIS functions: calculating coverage

Number of the question	Question	Answer	Overall		CAR								Total		Ethiopia										Total		Tanzania								Total		Uganda								Total		P-value by country
			N=53		Bangui City Administration (central/MOH) N=1		Health region 1 N=1		Health region 2 N=2		Health region 7 N=3		N= 7		Addis Ababa City Administration N=2		Addis Ababa City (central/MOH) N=1		Amhara and Gambella N=2		Oromia N=3		South Ethiopia and Sidama N=1		N=8		Dar es Salaam City Administration (central/MOH) N=1		Iringa N=6		Shinyanga N=5		Simiyu N=6		N=18		Kampala City Administration (central/MOH) N=1		Lango N=6		Karamoja N=8		West-Nile N=5		N=20		
			n	%	n	%	n	%	n	%	n	%	n	%	n	%	n	%	n	%	n	%	n	%	n	%	n	%	n	%	n	%	n	%	n	%	n	%	n	%	n	%	n	%	n	%	
ESF_014	Population estimates to calculate coverage	Yes	39	73.6	1	100	0	0	0	0	2	66.7	3	42.9	2	100	1	100	1	50	3	100	1	100	7	87.5	1	100	2	33.3	3	60	6	100	12	66.7	1	100	6	100	7	87.5	3	60	17	85	0.129
ESF015_1	Coverages for Antenatal care first visit	Yes	45	84.9	0	0	0	0	1	50	2	66.7	3	42.9	2	100	1	100	2	100	3	100	0	0	7	87.5	1	100	4	66.7	5	100	6	100	16	88.9	1	100	6	100	7	87.5	5	100	19	95	0.018
ESF015_2	Coverages for Deliveries at health facilities	Yes	47	88.7	0	0	0	0	1	50	3	100	4	57.1	2	100	1	100	2	100	3	100	0	0	7	87.5	1	100	5	83.3	5	100	6	100	17	94.4	1	100	6	100	7	87.5	5	100	19	95	0.049
ESF015_4	Coverages for Stillbirth rate in a health facility	Yes	41	77.4	0	0	0	0	0	0	2	66.7	2	28.6	2	100	1	100	2	100	3	100	1	100	8	100	1	100	0	0	5	100	6	100	12	66.7	1	100	6	100	7	87.5	5	100	19	95	0.001
ESF015_5	Coverages for Pre-discharge neonatal mortality rate	Yes	35	66	0	0	0	0	0	0	0	0	0	0	2	100	1	100	2	100	3	100	1	100	8	100	1	100	0	0	2	40	6	100	9	50	1	100	6	100	7	87.5	4	80	18	90	<0.001
ESF015_6	Coverages for Low birth weight rate among live births	Yes	38	71.7	0	0	0	0	0	0	1	33.3	1	14.3	2	100	1	100	2	100	1	33.3	1	100	6	75	1	100	0	0	5	100	6	100	12	66.7	1	100	6	100	7	87.5	5	100	19	95	0.001
ESF015_7	Coverages for Preterm birth (facility based)	Yes	38	71.7	0	0	0	0	0	0	1	33.3	1	14.3	1	50	1	100	2	100	2	66.7	1	100	6	75	0	0	4	66.7	2	40	6	100	12	66.7	1	100	6	100	7	87.5	5	100	19	95	0.001
ESF015_8	Coverages for Postnatal care for newborns (Facility-baed)	Yes	38	71.7	0	0	0	0	0	0	1	33.3	1	14.3	0	0	0	0	2	100	1	33.3	0	0	3	37.5	1	100	5	83.3	5	100	6	100	17	94.4	1	100	6	100	6	75	4	80	17	85	<0.001
ESF015_9	Coverages for Newborns with documented birthweight	Yes	41	77.4	0	0	0	0	0	0	1	33.3	1	14.3	0	0	0	0	2	100	2	66.7	0	0	4	50	1	100	5	83.3	5	100	6	100	17	94.4	1	100	6	100	7	87.5	5	100	19	95	<0.001
ESF015_10	Coverages for Newborns breastfed within one hour from birth	Yes	40	75.5	0	0	0	0	0	0	2	66.7	2	28.6	0	0	0	0	0	0	0	0	0	0	0	0	1	100	6	100	5	100	6	100	18	100	1	100	6	100	8	100	5	100	20	100	<0.001
ESF015_11	Coverages for Newborn resuscitation with bag and mask	Yes	46	86.8	0	0	0	0	0	0	1	33.3	1	14.3	2	100	1	100	2	100	3	100	0	0	7	87.5	1	100	6	100	5	100	6	100	18	100	1	100	6	100	8	100	5	100	20	100	<0.001
ESF015_12	Coverages for LBW babies initiating KMC	Yes	44	83	0	0	0	0	0	0	2	66.7	2	28.6	2	100	1	100	2	100	3	100	1	100	8	100	1	100	3	50	5	100	6	100	15	83.3	1	100	6	100	7	87.5	5	100	19	95	0.001
ESF015_13	Coverages for Newborns treated for sepsis/infection	Yes	43	81.1	0	0	0	0	0	0	1	33.3	1	14.3	2	100	1	100	2	100	1	33.3	1	100	6	75	1	100	6	100	4	80	6	100	17	94.4	1	100	6	100	7	87.5	5	100	19	95	<0.001
ESF015_14	Coverages for Antenatal corticosteroid use	Yes	19	35.8	0	0	0	0	0	0	0	0	0	0	0	0	0	0	0	0	0	0	1	100	1	12.5	0	0	4	66.7	4	80	0	0	8	44.4	1	100	4	66.7	4	50	1	20	10	50	0.041
ESF015_15	Coverages for Companion of choice during birth	Yes	4	7.5	0	0	0	0	0	0	0	0	0	0	0	0	0	0	0	0	0	0	0	0	0	0	0	0	0	0	0	0	0	0	0	0	0	0	2	33.3	2	25	0	0	4	20	0.130
ESF015_16	Coverages for Zero separation of mother and newborn	Yes	4	7.5	0	0	0	0	0	0	0	0	0	0	0	0	0	0	0	0	0	0	0	0	0	0	0	0	0	0	0	0	0	0	0	0	0	0	2	33.3	2	25	0	0	4	20	0.130
ESF015_17	Coverages for Respectful care indicator	Yes	3	5.7	0	0	0	0	0	0	0	0	0	0	0	0	0	0	0	0	0	0	0	0	0	0	0	0	0	0	0	0	0	0	0	0	0	0	2	33.3	1	12.5	0	0	3	15	0.319
ESF016_1	Available coverage measures at Region level	Yes	30	56.6	0	0	0	0	0	0	0	0	0	0	2	100	1	100	2	100	0	0	1	100	5	62.5	1	100	2	33.3	5	100	6	100	14	77.8	1	100	2	33.3	4	50	4	80	11	55	0.034
ESF016_2	Available coverage measures at District level	Yes	48	90.6	0	0	0	0	1	50	1	33.3	2	28.6	2	100	1	100	2	100	3	100	1	100	8	100	1	100	6	100	5	100	6	100	18	100	1	100	6	100	8	100	5	100	20	100	0.005
ESF016_3	Available coverage measures at health facility level	Yes	45	84.9	0	0	0	0	0	0	3	100	3	42.9	2	100	1	100	2	100	3	100	1	100	8		1	100	6	100	5	100	6	100	18	100	1	100	5	83.3	6	75	4	80	16	80	0.084

ESF016_4	Available coverage measures at Community level	Yes	12	22.6	0	0	0	0	0	0	1	33.3	1	14.3	1	50	1	100	2	100	1	33.3	1	100	5	0	0	0	0	0	0	0	0	0	0	0	0	2	33.3	2	25	2	40	6	30	0.002
ESF016_2-4	Available coverage measures at not regional level (District, HF or community)	Yes	50	94.3	0	0	0	0	1	50	3	100	4	57.1	2	100	1	100	2	100	3	100	1	100	8	1	100	6	100	5	100	6	100	18	100	1	100	6	100	8	100	5	100	20	100	>0.99

Notes: Indicators ESF015 are observed.

Abbreviations: CAR= Central African Republic; HF= health facility; KMC=kangaroo mother care; LBW=low birth weight; MOH=ministry of health

Appendix S8. Existing eRHIS functions: data integration (all OBSERVED)

Number of the question	Question	Answer	Overall		CAR								Total		Ethiopia										Total		Tanzania								Total		Uganda								Total		P-value by country
			N=53		Bangui City Administration (central/MOH) N=1		Health region 1 N=1		Health region 2 N=2		Health region 7 N=3		N= 7		Addis Ababa City Administration N=2		Addis Ababa City (central/MOH) N=1		Amhara and Gambella N=2		Oromia N=3		South Ethiopia and Sidama N=1		N=8		Dar es Salaam City Administration (central/MOH) N=1		Iringa N=6		Shinyanga N=5		Simiyu N=6		N=18		Kampala City Administration (central/MOH) N=1		Lango N=6		Karamoja N=8		West-Nile N=5		N=20		
			n	%	n	%	n	%	n	%	n	%	n	%	n	%	n	%	n	%	n	%	n	%	n	%	n	%	n	%	n	%	n	%	n	%	n	%	n	%	n	%	n	%	n	%	
ESF_017	Other software app (other than DHIS2)*	Yes	19	35.8	0	0	0	0	1	50	0	0	1	14.3	0	0	0	0	0	0	0	0	0	0	0	0	1	100	6	100	1	20	3	50	11	61.1	1	100	3	50	1	12.5	2	40	7	35	0.01
ESF_019	Degree of interoperability with parallel software (on the total sample, yes=yes or yes, partially)	Yes	8	15.1	0	0	0	0	0	0	0	0	0	0	0	0	0	0	0	0	0	0	0	0	0	0	1	100	1	16.7	0	0	0	0	2	11.1	0	0	3	50	1	12.5	2	40	6	30	0.013
ESF_020	RHIS integrate with HR info	Yes	4	7.5	0	0	0	0	0	0	0	0	0	0	0	0	0	0	0	0	0	0	1	100	1	12.5	0	0	0	0	0	0	0	0	0	0	0	0	1	16.7	2	25	0	0	3	15	0.303
ESF_021	RHIS integrate with logistic info	Yes	17	32.1	0	0	0	0	0	0	0	0	0	0	0	0	0	0	0	0	1	33.3	1	100	2	25	1	100	0	0	1	20	0	0	2	11.1	1	100	4	66.7	5	62.5	3	60	13	65	0.001
ESF_022	RHIS integrate with financial info	Yes	9	17	1	100	0	0	1	50	2	66.7	4	57.1	1	50	0	0	0	0	0	0	1	100	2	25	0	0	0	0	0	0	0	0	0	0	0	0	1	16.7	2	25	0	0	3	15	0.005
ESF_023	RHIS integrate with IDSR/notifiable diseases	Yes	34	64.2	1	100	0	0	2	100	2	66.7	5	71.4	1	50	0	0	1	50	1	33.3	1	100	4	50	1	100	5	83.3	0	0	3	50	9	50	1	100	4	66.7	6	75	5	100	16	80	0.209

Notes: All indicators in the table are observed; * the list of software app by country is reported in the following table.

Abbreviations: CAR= Central African Republic; DHIS2= District health information software, version 2; HR= human resources; IDSR = integrated disease surveillance and response; MOH=ministry of health

Appendix S9. Existing eRHIS functions: data disaggregation

Number of the question	Question	Answer	Overall		CAR								Total		Ethiopia										Total		Tanzania								Total		Uganda								Total		P-value by country
			N=53		Bangui City Administration (central/MOH) N=1		Health region 1 N=1		Health region 2 N=2		Health region 7 N=3		N= 7		Addis Ababa City Administration N=2		Addis Ababa City (central/MOH) N=1		Amhara and Gambella N=2		Oromia N=3		South Ethiopia and Sidama N=1		N=8		Dar es Salaam City Administration (central/MOH) N=1		Iringa N=6		Shinyanga N=5		Simiyu N=6		N=18		Kampala City Administration (central/MOH) N=1		Lango N=6		Karamoja N=8		West-Nile N=5		N=20		
			n	%	n	%	n	%	n	%	n	%	n	%	n	%	n	%	n	%	n	%	n	%	n	%	n	%	n	%	n	%	n	%	n	%	n	%	n	%	n	%	n	%	n	%	
ESF_024	Data disaggregation by age	Yes	36	67.9	0	0	0	0	0	0	1	33.3	1	14.3	1	50	1	100	2	100	2	66.7	1	100	6	75	1	100	3	50	2	40	4	66.7	10	55.6	1	100	6	100	7	87.5	5	100	19	95	<0.001
ESF_025	Data disaggregation by sex	Yes	40	75.5	0	0	0	0	0	0	1	33.3	1	14.3	2	100	1	100	2	100	2	66.7	0	0	6	75	1	100	2	33.3	5	100	6	100	14	77.8	1	100	6	100	7	87.5	5	100	19	95	<0.001
ESF_025.1	Data disaggregation by socioeconomic status	Yes	5	9.4	0	0	0	0	0	0	0	0	0	0	1	50	1	100	0	0	1	33.3	0	0	2	25	0	0	0	0	0	0	0	0	0	0	0	0	2	33.3	1	12.5	0	0	3	15	0.130
ESF_025.2	Data disaggregation by maternal education	Yes	2	3.8	0	0	0	0	0	0	0	0	0	0	0	0	0	0	0	0	0	0	0	0	0	0	0	0	0	0	0	0	0	0	0	0	0	0	1	16.7	0	0	1	20	2	10	0.739
ESF_025.3	Data disaggregation by urban/rural	Yes	16	30.2	0	0	0	0	0	0	0	0	0	0	2	100	1	100	1	50	1	33.3	1	100	5	62.5	0	0	1	16.7	0	0	0	0	1	5.6	0	0	2	33.3	5	62.5	3	60	10	50	0.001
ESF_025.4	Data disaggregation by other determinants	Yes	11	20.8	0	0	0	0	0	0	0	0	0	0	2	100	1	100	0	0	2	66.7	1	100	5	62.5	0	0	0	0	0	0	0	0	0	0	1	100	0	0	4	50	1	20	6	30	0.001

Notes: All indicators in the table are observed.

Abbreviations: CAR= Central African Republic; MOH=ministry of health

Appendix S10. Existing eRHIS functions: unique identifier

Number of the question	Question	Answer	Overall		CAR								Total		Ethiopia										Total		Tanzania								Total		Uganda								Total		P-value by country
			N=53		Bangui City Administration (central/MOH) N=1		Health region 1 N=1		Health region 2 N=2		Health region 7 N=3		N= 7		Addis Ababa City Administration N=2		Addis Ababa City (central/MOH) N=1		Amhara and Gambella N=2		Oromia N=3		South Ethiopia and Sidama N=1		N=8		Dar es Salaam City Administration (central/MOH) N=1		Iringa N=6		Shinyanga N=5		Simiyu N=6		N=18		Kampala City Administration (central/MOH) N=1		Lango N=6		Karamoja N=8		West-Nile N=5		N=20		
			n	%	n	%	n	%	n	%	n	%	n	%	n	%	n	%	n	%	n	%	n	%	n	%	n	%	n	%	n	%	n	%	n	%	n	%	n	%	n	%	n	%	n	%	
ESF_026.1	Master Facility List	Yes	38	71.7	1	100	0	0	0	0	1	33.3	2	28.6	2	100	1	100	2	100	2	66.7	1	100	7	87.5	1	100	4	66.7	2	40	6	100	13	72.2	1	100	4	66.7	6	75	5	100	16	80	0.059
ESF_026.1&2	Master or built-in facility list used	Yes	42	79.2	1	100	0	0	1	50	2	66.7	4	57.1	2	100	1	100	2	100	3	100	1	100	8	100	1	100	4	66.7	3	60	6	100	14	77.8	1	100	4	66.7	6	75	5	100	16	80	0.237
ESF_028	Geographic coordinates attached (yes=1-100%facilities)	Yes	33	62.3	1	100	0	0	0	0	1	33.3	2	28.6	0	0	0	0	1	50	3	100	0	0	4	50	1	100	6	100	2	40	0	0	9	50	1	100	6	100	6	75	5	100	18	90	0.005
ESF_029	Unique identifiers for district and regions in RHIS	Yes	37	69.8	1	100	0	0	0	0	0	0	1	14.3	2	100	1	100	2	100	3	100	1	100	8	100	1	100	5	83.3	1	20	6	100	13	72.2	1	100	6	100	5	62.5	3	60	15	75	0.003

Notes: All indicators in the table are observed.
Abbreviations: CAR= Central African Republic; MFL= Master Facility List; MOH=ministry of health

Appendix S11. Existing eRHIS functions: data visualization

Number of the question	Question	Answer	Overall		CAR								Total		Ethiopia										Total		Tanzania								Total		Uganda								Total		P-value by country
			N=53		Bangui City Administration (central/MOH) N=1		Health region 1 N=1		Health region 2 N=2		Health region 7 N=3		N= 7		Addis Ababa City Administration N=2		Addis Ababa City (central/MOH) N=1		Amhara and Gambella N=2		Oromia N=3		South Ethiopia and Sidama N=1		N=8		Dar es Salaam City Administration (central/MOH) N=1		Iringa N=6		Shinyanga N=5		Simiyu N=6		N=18		Kampala City Administration (central/MOH) N=1		Lango N=6		Karamoja N=8		West-Nile N=5		N=20		
			n	%	n	%	n	%	n	%	n	%	n	%	n	%	n	%	n	%	n	%	n	%	n	%	n	%	n	%	n	%	n	%	n	%	n	%	n	%	n	%	n	%	n	%	
	1. Data visualization by type of indicator																																														
ESF031_1	A. Mortality neonatal rate																																														
ESF032	RHIS generates tables	Yes	48	90.6	1	100	0	0	0	0	3	100	4	57.1	2	100	1	100	2	100	3	100	1	100	8	100	1	100	4	66.7	5	100	6	100	16	88.9	1	100	6	100	8	100	5	100	20	100	0.006
ESF033	RHIS generates time trends graphs	Yes	48	90.6	1	100	0	0	0	0	3	100	4	57.1	2	100	1	100	2	100	3	100	1	100	8	100	1	100	4	66.7	5	100	6	100	16	88.9	1	100	6	100	8	100	5	100	20	100	0.006
ESF034	Visualization by subgroups (both facility and district regions)	Yes	47	88.7	1	100	0	0	0	0	1	33.3	2	28.6	2	100	1	100	2	100	3	100	1	100	8	100	1	100	5	83.3	5	100	6	100	17	94.4	1	100	6	100	8	100	5	100	20	100	<0.001
ESF036.5	RHIS report on major causes of mortality	Yes	29	54.7	0	0	0	0	0	0	0	0	0	0	0	0	0	0	0	0	0	0	0	0	0	0	1	100	4	66.7	1	20	4	66.7	10	55.6	1	100	6	100	8	100	4	80	19	95	<0.001
ESF037.5	RHIS report on major causes of morbidity	Yes	28	52.8	0	0	0	0	0	0	0	0	0	0	0	0	0	0	0	0	0	0	0	0	0	0	1	100	4	66.7	1	20	4	66.7	10	55.6	1	100	6	100	7	87.5	4	80	18	90	<0.001
ESF031_2	B. Stillbirth rate																																														
ESF032	RHIS generates tables	Yes	48	90.6	1	100	0	0	0	0	3	100	4	57.1	2	100	1	100	2	100	3	100	1	100	8	100	1	100	4	66.7	5	100	6	100	16	88.9	1	100	6	100	8	100	5	100	20	100	0.006
ESF033	RHIS generates time trends graphs	Yes	48	90.6	1	100	0	0	0	0	3	100	4	57.1	2	100	1	100	2	100	3	100	1	100	8	100	1	100	4	66.7	5	100	6	100	16	88.9	1	100	6	100	8	100	5	100	20	100	0.006
ESF034	Visualization by subgroups (both facility and district regions)	Yes	47	88.7	1	100	0	0	0	0	1	33.3	2	28.6	2	100	1	100	2	100	3	100	1	100	8	100	1	100	5	83.3	5	100	6	100	17	94.4	1	100	6	100	8	100	5	100	20	100	<0.001
ESF031_3	C. Low birthweigh rate																																														
ESF032	RHIS generates tables	Yes	46	86.8	1	100	0	0	0	0	3	100	4	57.1	2	100	1	100	2	100	1	33.3	1	100	6	75	1	100	4	66.7	5	100	6	100	16	88.9	1	100	6	100	8	100	5	100	20	100	0.011
ESF033	RHIS generates time trends graphs	Yes	46	86.8	1	100	0	0	0	0	3	100	4	57.1	2	100	1	100	2	100	1	33.3	1	100	6	75	1	100	4	66.7	5	100	6	100	16	88.9	1	100	6	100	8	100	5	100	20	100	0.011
ESF034	Visualization by subgroups (both facility and district regions)	Yes	45	84.9	1	100	0	0	0	0	1	33.3	2	28.6	2	100	1	100	2	100	1	33.3	1	100	6	75	1	100	5	83.3	5	100	6	100	17	94.4	1	100	6	100	8	100	5	100	20	100	<0.001
ESF031_4	D. Kangaroo Mother Care initiation																																														
ESF032	RHIS generates tables	Yes	46	86.8	1	100	0	0	0	0	3	100	4	57.1	2	100	1	100	1	50	3	100	1	100	7	87.5	1	100	3	50	5	100	6	100	15	83.3	1	100	6	100	8	100	5	100	20	100	0.022
ESF033	RHIS generates time trends graphs	Yes	46	86.8	1	100	0	0	0	0	3	100	4	57.1	2	100	1	100	1	50	3	100	1	100	7	87.5	1	100	3	50	5	100	6	100	15	83.3	1	100	6	100	8	100	5	100	20	100	0.022
ESF034	Visualization by subgroups (both facility and district regions)	Yes	45	84.9	1	100	0	0	0	0	1	33.3	2	28.6	2	100	1	100	1	50	3	100	1	100	7	87.5	1	100	4	66.7	5	100	6	100	16	88.9	1	100	6	100	8	100	5	100	20	100	<0.001

**ESF031_5**	**E. Early initiation of breastfeeding**																																														
ESF032	RHIS generates tables	Yes	40	75.5	1	100	0	0	0	0	3	100	4	57.1	0	0	0	0	0	0	0	0	0	0	0	0	1	100	4	66.7	5	100	6	100	16	88.9	1	100	6	100	8	100	5	100	20	100	< 0.001
ESF033	RHIS generates time trends graphs	Yes	40	75.5	1	100	0	0	0	0	3	100	4	57.1	0	0	0	0	0	0	0	0	0	0	0	0	1	100	4	66.7	5	100	6	100	16	88.9	1	100	6	100	8	100	5	100	20	100	< 0.001
ESF034	Visualization by subgroups (both facility and district regions)	Yes	39	73.6	1	100	0	0	0	0	1	33.3	2	28.6	0	0	0	0	0	0	0	0	0	0	0	0	1	100	5	83.3	5	100	6	100	17	94.4	1	100	6	100	8	100	5	100	20	100	< 0.001
	2. Data visualization by thematic map																																														
ESF035	RHIS allowing visualization by thematic map	By region	37	69.8	1	100	0	0	0	0	0	0	1	14.3	0	0	0	0	2	100	0	0	1	100	3	37.5	1	100	3	50	5	100	6	100	15	83.3	1	100	6	100	6	75	5	100	18	90	<0.001
ESF035	Visualization by thematic map	By district	44	83	1	100	0	0	1	50	0	0	2	28.6	0	0	0	0	2	100	2	66.7	1	100	5	62.5	1	100	5	83.3	5	100	6	100	17	94.4	1	100	6	100	8	100	5	100	20	100	<0.001
ESF035	Visualization by thematic map	By facility	43	81.1	1	100	0	0	1	50	3	100	5	71.4	0	0	0	0	2	100	1	33.3	0	0	3	37.5	1	100	5	83.3	5	100	6	100	17	94.4	1	100	6	100	7	87.5	4	80	18	90	0.005
ESF035	Visualization by thematic map	By community-level SDP	19	35.8	1	100	0	0	0	0	0	0	1	14.3	0	0	0	0	2	100	0	0	0	0	2	25	1	100	2	33.3	0	0	0	0	3	16.7	1	100	6	100	4	50	2	40	13	65	0.007

Notes: All indicators in the table are observed.

Abbreviations: CAR= Central African Republic; MOH=ministry of health; SDP = service delivery point

Appendix S12. End-Users' perspectives on need for improvement in eRHIS

A) Figure: End-users' perspectives on need for improvement in eRHIS (N=53)

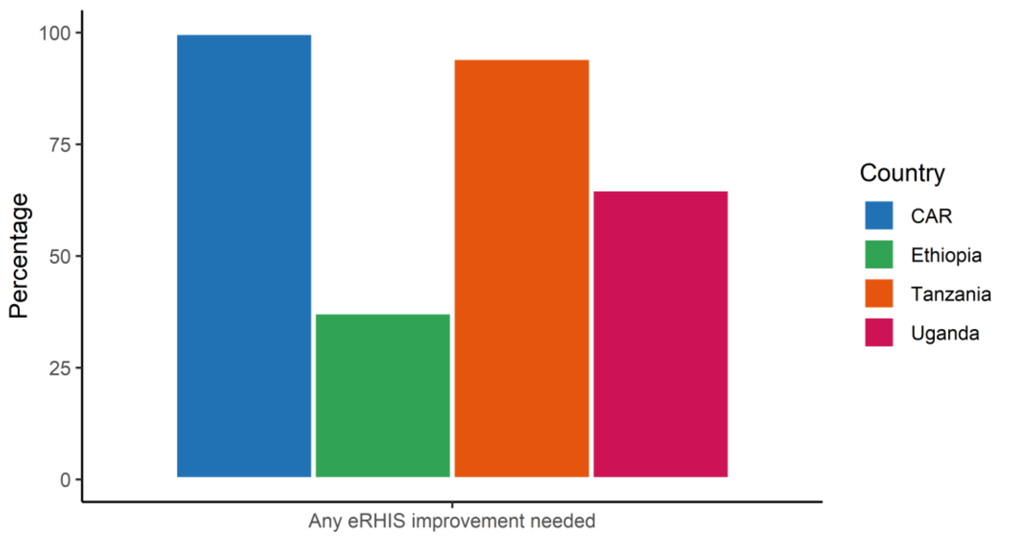

Abbreviations: CAR= Central African Republic; eRHIS=electronic Routine Health information System

B) Table: End-users' perspectives on need for improvement in eRHIS

Question number	Answer	Overall N=53		CAR: Bangui City Administration (central/MOH) N=1		CAR: Health region 1 N=1		CAR: Health region 2 N=2		CAR: Health region 7 N=3		CAR Total N= 7	
		n	%	n	%	n	%	n	%	n	%	n	%
ESF_110.1.1	Any improvement needed (some+major)	40	75.5	1	100	1	100	2	100	3	100	7	100
	No improvement needed	13	24.5	0	0	0	0	0	0	0	0	0	0
	Some improvement needed	31	58.5	1	100	1	100	2	100	1	33.3	5	71.4
	Major improvement needed	9	17	0	0	0	0	0	0	2	66.7	2	28.6

Question number	Answer	Ethiopia: Addis Ababa City Administration N=2		Ethiopia: Addis Ababa City (central/MOH) N=1		Ethiopia: Amhara and Gambella N=2		Ethiopia: Oromia N=3		Ethiopia: South Ethiopia and Sidama N=1		Ethiopia Total N=8	
		n	%	n	%	n	%	n	%	n	%	n	%
ESF_110.1.1	Any improvement needed (some+major)	2	100	1	100	0	0	1	33.3	0	0	3	37.5
	No improvement needed	0	0	0	0	2	100	2	66.7	1	100	5	62.5
	Some improvement needed	0	0	0	0	0	0	1	33.3	0	0	1	12.5
	Major improvement needed	2	100	1	100	0	0	0	0	0	0	2	25

Question number	Answer	Tanzania: Dar es Salaam City Administration (central/MOH) N=1		Tanzania: Iringa N=6		Tanzania: Shinyanga N=5		Tanzania: Simiyu N=6		Tanzania Total N=18	
		n	%	n	%	n	%	n	%	n	%
ESF_110.1.1	Any improvement needed (some+major)	1	100	6	100	4	80	6	100	17	94.4
	No improvement needed	0	0	0	0	1	20	0	0	1	5.6
	Some improvement needed	1	100	3	50	4	80	6	100	14	77.8
	Major improvement needed	0	0	3	50	0	0	0	0	3	16.7

Question number	Answer	Uganda: Kampala City Administration (central/MOH) N=1		Uganda: Lango N=6		Uganda: Karamoja N=8		Uganda: West-Nile N=5		Uganda Total N=20		P-value by country
		n	%	n	%	n	%	n	%	n	%	
ESF_110.1.1	Any improvement needed (some+major)	1	100	3	50	4	50	5	100	13	65	0.004
	No improvement needed	0	0	3	50	4	50	0	0	7	35	0.007
	Some improvement needed	1	100	3	50	3	37.5	4	80	11	55	
	Major improvement needed	0	0	0	0	1	12.5	1	20	2	10	

Abbreviations: CAR= Central African Republic; eRHIS=electronic Routine Health information System; MOH=ministry of health
